# Active night-time tweeting is associated with meaningfully lower mental wellbeing in a UK birth cohort study

**DOI:** 10.1038/s41598-025-14745-y

**Published:** 2025-10-09

**Authors:** Daniel Joinson, Claire M. A. Haworth, Edwin Simpson, Nello Cristianini, Nina H. Di Cara, Oliver S. P. Davis

**Affiliations:** 1 School of Engineering Mathematics and Technology, University of Bristol, Bristol, UK; 2 School of Psychological Science, University of Bristol, Bristol, UK; 3 Department of Computer Science, University of Bath, Bath, UK; 4 Department of Population Health Sciences (Bristol Medical School), MRC Integrative Epidemiology Unit, University of Bristol, Bristol, UK

**Keywords:** Epidemiology, Human behaviour, Statistics, Anxiety, Depression

## Abstract

It has been suggested that use of social media late at night could lead to worse mental health outcomes. We linked Twitter (‘X’) data to self-reported measures of mental health from the Avon Longitudinal Study of Parents and Children. We aimed to predict these measures from the average hour a participant posted Tweets. We used data from 310 adult participants and 18,288 Tweets. We found strong evidence the average hour participants posted Tweets was associated with depressive symptoms, anxiety symptoms, and mental wellbeing. Average hour explained almost 2% of the variation in mental wellbeing, comparable to reports of the impact of binge drinking and exercise. Participants who, on average, Tweeted through the night (23:00 to 05:00) showed meaningfully worse mental wellbeing than those who Tweeted during the daytime. Although the average hour that a participant posted their Tweets explained less of the variation in their depressive (0.2%) and anxiety (0.7%) symptoms, after stratification by age and sex these relationships became stronger. Our results could inform behavioural interventions for improving the safety of social media platforms.

**Supplementary Information:**

The online version contains supplementary material available at 10.1038/s41598-025-14745-y.

## Introduction

Due to the scale and intensity of social media use, any positive or negative effect on mental health would impact a substantial number of people worldwide. Indeed, improving the safety of social media platforms is a global concern, as evidenced by a surge in legislative and regulatory measures across the world, including the UK Online Safety Bill, EU Digital Services Act and proposed US Platform Accountability and Transparency Act^1–3^. While legislation should be supported by evidence, in the case of social media use and mental health the evidence base is contradictory and incomplete.

Most previous research has explored the relationship between the *frequency* of social media use and mental health. Several systematic reviews and meta-analyses have found evidence for a small association between greater frequency of social media use and worse mental health outcomes^4–9^. These results are supported by an analysis conducted on three large-scale datasets from the USA and UK (total *N* = 355,358), which conclude that social media has a trivial average effect on the mental health of adolescents^10^. This is challenged by another study, which repeated this analysis whilst changing several aspects of the methodology^11^. This study found evidence that the negative effect of social media in females was comparable to risk factors such as binge drinking, obesity and drug use^11^. Other reviews have found evidence that social media has a positive effect on mental health^12^.

These inconsistencies could be explained by the focus on measuring the frequency individuals use social media. Frequency does not capture the wide range of behaviours that can be performed within these sites^13,14^. For example, two individuals could use social media for an identical amount of time, but perform completely different behaviours^13,14^. These behaviours could have very different implications for their mental health. In addition, the impact of social media on mental health may differ based on the characteristics of users (e.g. age, sex). To fully understand the relationship between social media and mental health, we need a more nuanced focus on exploring specific behaviours and types of users. Identifying behaviours that are beneficial or problematic for mental health, and the users most at risk, will provide strong and relevant evidence to improve the safety of social media platforms.

### Nighttime use of social media

Some previous studies have focused on more specific behaviours, for example the use of social media at nighttime. Nighttime use of social media could displace sleep, as individuals stay awake to use it^15–17^. Performing active behaviours like posting and messaging at nighttime could cause cognitive arousal, and blue light emitted by smartphones could inhibit the production of the hormone melatonin^15–18^. All these factors may combine to delay sleep onset, and lead to worse sleep quality and quantity. Improved sleep-related outcomes are in turn associated with better mental health^19^. Previous studies have found evidence that greater self-reported night time social media or technology use is associated with worse depressive symptoms and self-esteem^20–27^.

These studies captured social media behaviours through questionnaires that participants completed. However, there is evidence that self-reported measures of social media are not accurate^28–32^. It has been found that participants overestimate the amount they use social media, ranging from 51 min to 256 min per day^30^. This is a major limitation, and one which can be addressed by using data collected directly from social media sites. This provides accurate information about the amount of time individuals spent on social media and what behaviours they perform. For Twitter (now ‘X’), a few previous studies have used this data to explore the relationship between the time of day individuals post content (Tweets) and inferred mental health. These studies can be grouped in two categories, based on how they estimate the mental health status of their participants. Some aim to identify users with mental health conditions based on keywords and phrases used in their Tweets^33,34^. These users are then compared to random samples of Twitter users, who are assumed to not have the condition^33,34^. Such studies have found evidence that depressed users are more active on Twitter at nighttime^33,34^. The limitation of this approach is that it cannot verify the true clinical state of users in the sample, which could lead to actually depressed users being in the random sample and vice versa^35^. The second approach collects Tweets from a sample of recruited participants and estimates their mental health status through validated self-reported questionnaires^35^. For instance, one study classified participants into depressed and non-depressed based on responses to the Center for Epidemiologic Studies Depression Scale, finding that depressed participants were more active on Twitter at late night^36^. These studies generally treat their measures of mental health as binary variables. However, mental health conditions are fundamentally quantitative traits, and treating them as binary variables prevents us from exploring the full range of their variation^37^. Treating mental health conditions as continuous variables would allow us to quantify the relationship between the timing of Twitter activity and mental health. Only one Twitter study has done this, and found evidence that a higher proportion of Tweets posted at nighttime (21:00 to 06:00) was associated with more self-reported depressive symptoms^38^. None of the described studies considered how demographic covariates (e.g. sex, age) may influence the relationship they found, and most only measured depression status as their mental health outcome.

It is important that new studies build upon this evidence base, given public and political interest in the impact of nighttime social media use on mental health. Such evidence could inform legislation or interventions targeting this specific behaviour. For example, in 2023, the Chinese cyberspace regulator proposed legislation prohibiting the use of social media between 10 p.m. and 6 a.m. for under 18 year olds^39^. More recently, TikTok has introduced a tool called “Wind Down”, which automatically activates after 10pm for users under the age of 16^40^. This tool replaces the platform’s “For You” home page with calming music and breathing exercises^40^. The application of such a curfew to other social media platforms is being looked at by the UK Secretary of State for Science, Innovation and Technology, Peter Kyle^40^. These interventions aim to alter the social media behaviour of populations, although other approaches could focus on less systemic change^41^. Platforms could introduce tools that allow users to block themselves from using their site at nighttime, or prevent them from performing particular behaviours at this time (e.g. writing posts, messaging other users). Platforms could also be required to introduce informational alerts on their sites, to make users aware of the potential risks of nighttime use of social media.The purpose of our study is to inform the development of such legislation or interventions, by strengthening the evidence base we have described above. The main aim of our study is to investigate the relationship between the time of day Twitter users post on this platform and their mental health. We combined directly accessed Twitter data linked to participants in an epidemiological sample with high-quality measures of mental health and demographic information. We used a novel application of circular statistics to measure the average time a participant posted their Tweets, which avoids losing key information and can explore the relationship with mental health at a finer scale than previous studies. We built regression models to explore and quantify the relationship between the average hour a participant posted Tweets at and their symptoms of depression, anxiety and mental wellbeing. Because we collected these data in a well-characterised population cohort, we were also able to explore how these associations are influenced by the sex and age of participants.

## Methodology

### Sample

We used data from the Avon Longitudinal Study of Children and Parents (ALSPAC). This is a longitudinal cohort study, containing two-generations of participants who have been followed up through questionnaires and other forms of measurement for over three decades^42–45^. ALSPAC began with the recruitment of pregnant women, from the Avon area of the UK, who had expected dates of delivery between 1st April 1991 and 31st December 1992^42–46^. 14,541 pregnancies were enrolled in the study, resulting in 13,988 children who were alive at 1 year of age^42–46^. Further phases of recruitment, involving eligible but unenrolled pregnancies, resulted in a sample size of 15,447 pregnancies^42–46^. This consists of 14,833 mothers and 14,901 children alive at 1 year of age^42–46^. In addition, the partners of the mothers were invited to enrol in the study, resulting in a sample of 3807 partners^42–46^. Our study uses data from these two generations with ALSPAC: original pregnant women, biological fathers and other carers/partners (G0 sample), and index children (G1 sample)^42–46^.

Data used in our study were collected and managed using REDCap electronic data capture tools hosted at the University of Bristol^47^. REDCap (Research Electronic Data Capture) is a secure, web-based software platform designed to support data capture for research studies [74]. The ALSPAC study website contains details of all the data that is available through a fully searchable data dictionary and variable search tool^48^. Ethical approval for our study was obtained from the ALSPAC Ethics and Law Committee and the Local Research Ethics Committees, with all methods performed in accordance with their relevant guidelines and regulations. Informed consent for the use of data collected via questionnaires and clinics was obtained from participants following the recommendations of the ALSPAC Ethics and Law Committee at the time^49^.

### Data collection

We used two different types of data from ALSPAC. First, self-reported survey data from five questionnaires (C1,2,4,5 and 6) completed by G0 and G1 participants between April 2020 and May 2022. We used data from these five questionnaires in order to increase the number of observations our models were fitted with. In addition, all G0 and G1 participants were invited to participate in the Twitter data linkage. 623 participants were successfully linked, with their Twitter data harvested using the Epicosm software, which was created specifically to facilitate the collection and linkage of Twitter data in epidemiological cohorts^50^. With their consent, we used the Twitter V1.1 academic API to harvest the entire timeline of the 623 participants. This includes the Tweets and re-tweets posted by participants, alongside metadata about these posts (e.g. time posted, date posted, number of likes). The Twitter data were anonymised prior to being made available to researchers, in order to prevent the participants being identified. All Tweet text data were removed and the time of posting was represented with 1-hour time windows. This Twitter data contained 1,488,517 Tweets posted between January 2008 and February 2023.

### Measures

#### Depression, anxiety and mental wellbeing

Depressive symptoms were measured using the Short Mood and Feelings Questionnaire (SMFQ). This is a 13-item self-report measure of depressive symptoms over the past 2 weeks^51^. The SMFQ ranges between 0 and 26, with higher scores representing more depressive symptoms^51^.

Anxiety symptoms were measured using the seven-item Generalized Anxiety Disorder scale 2 (GAD-7), which was developed to screen for generalised anxiety disorder and assess symptom severity^52^. The GAD-7 asks participants to report the presence of anxiety-related problems over the last 2 weeks. The GAD-7 ranges between 0 and 21, with higher scores indicating worse anxiety symptoms.

Mental wellbeing was measured using the Warwick Edinburgh Mental Wellbeing Scale (WEMWBS). This is a measure of mental wellbeing in the general population, containing positively worded questions covering areas such as feelings and functioning^53^. This study used the 14-item scale, which asks respondents how often they experienced particular feelings in the past 2 weeks^53^. The WEMWBS scores range between 14 and 70, with higher scores indicating better mental wellbeing.

Table 1 shows the start and end date of data collection for each questionnaire, and whether it contained the three mental health measures.

**Table 1:** ALSPAC questionnaires used in this study.

Questionnaire	Start date	End date	SMFQ	GAD-7	WEMWBS
C1	09/04/2020	14/05/2020	X	X	X
C2	26/05/2020	05/07/2020	X	X	X
C4	17/11/2020	07/02/2021	X	X	X
C5	21/06/2021	22/12/2021	X	X	
C6	29/04/2022	12/05/2022	X	X	X

#### Average hour of tweet posting

A common approach used to quantify the time of day an individual posts their Tweet involves grouping hours into two windows: daytime and nighttime^36,38^. Previous studies have used time windows of 06:01 to 20:59 for daytime and 21:00 to 06:00 for nighttime^36,38^. This approach enables useful broad comparisons, but results in a loss of information and ability to find finer-scale relationships. For example, this approach would treat a participant who posted all their Tweets at 21:30 and one who posted all their Tweets at 04:00 as identical. New methodologies are required that can summarise the time participants post Tweets without losing key information.

A challenge of summarising the hour at which a Tweet is posted is that, in the 24-hour clock, times that appear numerically far apart, such as 00:05 and 23:55, are actually close together. This challenge can be addressed by modelling the time the Tweet was posted as a position on a circle representing the hour of the day, with each position expressed by a combination of the sine and cosine of the angle. We calculated the average hour each participant posted their Tweets using this circular representation, as described in Supplementary Materials. Our approach produces a single variable representing the mean time of day each participant posted their Tweets at, without losing information through grouping hours into time windows.

#### Covariates

The sex and ethnicity of G0 and G1 participants was reported during the mother’s pregnancy with the index child. The generation and age of each participant is also available. In 2020, when collection of the Twitter data started, the mean age of G0 participants was 56.27 and G1 was 27.47.

### Data preparation and inclusion criteria

Any of the 623 participants who provided Twitter data were eligible for analysis in our study if they provided a mental health measure in at least one of the five questionnaire, and they posted at least one Tweet in the 2-weeks before this questionnaire was completed. This resulted in a sample size of 310 participants. For each participant, we calculated the average hour at which they posted Tweets in the 2-week period before they completed that questionnaire. 2-weeks was chosen because it is the time period that the SMFQ, GAD-7 and WEMWBS measures aim to capture mental health or wellbeing status. This resulted in a dataset where each row was a single participant and their SMFQ score, GAD-7 score, WEMWBS score and average hour of Tweet posting from a single questionnaire. Thus, each participant can contribute up to five rows of data. We also calculated the total number of Tweets posted by each participant in this 2-week period.

We applied no other inclusion or exclusion criteria to our sample. As we were using secondary data, we did not perform a sample size calculation, and instead aimed to include as many participants as possible who fulfilled the inclusion criteria. Since ALSPAC is a multi-generational cohort study, some participants in our sample may belong to the same family. In total, 18 participants, representing 9 families, shared a familial relationship.

### Analysis

#### Unadjusted models

As the prepared dataset could contain multiple observations from the same participant or questionnaire, mixed effect modelling was required. The use of mixed effect models allowed us to include crossed random effects which accounted for variability explained by the structure of our dataset. In our dataset, these captured which participant and questionnaire an observation was from. The outcome in these models was either SMFQ, GAD-7 or WEMWBS score. The fixed effects were four trigonometric terms representing the mean hour of Tweet posting. Average hours of Tweet posting were converted into their angle form $\:\left(\stackrel{-}{\theta\:}\right)$ prior to their inclusion in the models, by dividing the hour by 24 and multiplying by 2$\:\pi\:$. Using model specification format from R, our mixed effect models had the following equation (EQ):

1 $$\:Outcome\:\sim\text{cos}\left(\stackrel{-}{\theta\:}\right)+\text{sin}\left(\stackrel{-}{\theta\:}\right)+\text{cos}\left(2\stackrel{-}{\theta\:}\right)+\text{sin}\left(2\stackrel{-}{\theta\:}\right)+\left(1|Participants\right)+(1\left|Questionnaire\right)$$

This base model is described as the unadjusted model. No additional variables (i.e. covariates) were included in this unadjusted model.

#### Adjusted models

Building upon the unadjusted model, we added additional fixed effect terms which controlled for the age and sex of participants. We coded sex and age as binary (− 1 or 1) variables, representing whether the participant was male or female, and in ALSPAC cohort G0 or G1. These were the only variables we adjusted for in this model. This covariate model had the R format:

2 $$\:Outcome\:\sim\text{cos}\left(\stackrel{-}{\theta\:}\right)+\text{sin}\left(\stackrel{-}{\theta\:}\right)+\text{cos}\left(2\stackrel{-}{\theta\:}\right)+\text{sin}\left(2\stackrel{-}{\theta\:}\right)+sex+generation+\:\left(1|Participants\right)+(1\left|Questionnaire\right)$$

#### Interaction models

We then produced models which allowed an interaction between the circular terms and participant’s demographic characteristics: (1) Interaction between circular terms and sex, (2) Interaction between circular terms and generation, and (3) Interaction between circular terms and both sex and generation. The purpose of these interaction terms was to explore how age and generation affect the relationship between average hour of Tweet posting and mental health. All of these interaction models were adjusted for the generation and sex of our participants. These models had the following R format:

3 $$\begin{aligned} & Outcome\:\sim ({\text{cos}}\left( {\mathop {\theta \:}\limits^{ - } } \right)*sex) + ({\text{sin}}\left( {\mathop {\theta \:}\limits^{ - } } \right)*sex) + ({\text{cos}}\left( {2\mathop {\theta \:}\limits^{ - } } \right)*sex) \\ & \quad + ({\text{sin}}\left( {2\mathop {\theta \:}\limits^{ - } } \right)*sex) + generation + \:\left( {1|Participants} \right) + (1|Questionnaire) \\ \end{aligned}$$

4 $$\begin{aligned} & Outcome\:\sim ({\text{cos}}\left( {\mathop {\theta \:}\limits^{ - } } \right)*generation) + ({\text{sin}}\left( {\mathop {\theta \:}\limits^{ - } } \right)*generation) + ({\text{cos}}\left( {2\mathop {\theta \:}\limits^{ - } } \right)*generation) \\ & \quad + \left( {{\text{sin}}\left( {2\mathop {\theta \:}\limits^{ - } } \right)*generation} \right) + sex + \:\left( {1|Participants} \right) + (1|Questionnaire) \\ \end{aligned}$$

5 $$\begin{aligned} & Outcome\:\sim ({\text{cos}}\left( {\mathop {\theta \:}\limits^{ - } } \right)*sex) + ({\text{sin}}\left( {\mathop {\theta \:}\limits^{ - } } \right)*sex) + ({\text{cos}}\left( {2\mathop {\theta \:}\limits^{ - } } \right)*sex) \\ & \quad + ({\text{sin}}\left( {2\mathop {\theta \:}\limits^{ - } } \right)*sex) + \:({\text{cos}}\left( {\mathop {\theta \:}\limits^{ - } } \right)*generation) + ({\text{sin}}\left( {\mathop {\theta \:}\limits^{ - } } \right)*generation) \\ & \quad + ({\text{cos}}\left( {2\mathop {\theta \:}\limits^{ - } } \right)*generation) + ({\text{sin}}\left( {2\mathop {\theta \:}\limits^{ - } } \right)*generation) + \left( {1|Participants} \right) + (1|Questionnaire) \\ \end{aligned}$$

#### Model analysis

Each model was weighted by the number of Tweets posted by the participant during the specified two-week period, after $\:log\:\left(x+1\right)\:\:$transformation. This transformation was necessary to avoid overfitting to participants who posted very large numbers of Tweets. We used linear regression for outcome variables that were normally distributed, and Poisson regression for non-normally distributed outcome variables. Our models were fitted using maximum-likelihood.

The unadjusted model (Eq. 1) was nested within the adjusted model (Eq. 2), which in turn was nested within the interaction models (Eqs. 3–5). As a result, the impact of adding new variables to models can be evaluated using likelihood ratio tests (LRTs). To test whether the average time participants posted Tweets was associated with a mental health outcome, the unadjusted and adjusted models were compared using LRTs to their null models, in which any circular terms were removed. To test whether there was evidence for an interaction between average hour of Tweet posting and the covariates, the interaction models were compared to the adjusted model.

For each model, we generated marginal R2 scores, which refer to the amount of variation in the outcome explained by the fixed effects alone. We also calculated partial R2 scores, to capture the amount of variation explained specifically by the average hour of Tweet posting. We generated Akaike information criteria (AIC) and Bayesian information criterion (BIC) scores for each model, to further assess model performance. Using results from the best fitting model, we generated and plotted predicted outcome values across the entire range of average hours of Tweet posting.

If there was sufficient evidence from the LRTs that the sex or generation interaction terms improved model fit, we produced stratified models. We repeated the previous analysis (i.e. LRTs, R2 scores, plotting predicted values), and compared these results across levels of the strata.

## Results

### Participant and tweet characteristics

623 ALSPAC participants provided Twitter data. The demographic characteristics of these participants are summarised in Table 2. These are compared to the characteristics of the wider ALSPAC cohort and the participants included in our models.

**Table 2:** Demographic characteristics of study participants and the wider ALSPAC cohort.

Demographic Variable	Linked Twitter Cohort (*N* = 623)	Wider ALSPAC Cohort (*N* = 33,419)	Sample used in regression models (*N* = 310)
Generation % (n/N)			
G0 (mothers)	21% (130/623)	44% (14,779/33,419)	20% (62/310)
G0 (partners)	15% (93/623)	11% (3761/33,419)	18% (56/310)
G1	64% (400/623)	45% (14,859/33,419)	62% (192/310)
Female % (n/N)			
G0	58% (130/223)	80% (14,799/18,560)	53% (62/118)
G1	66% (263/400)	49% (7266/14,859)	66% (127/192)
Mean Age (SD) in 2020			
G0	57.75 (4.89)	56.27 (5.33)	58.03 (4.85)
G1	27.52 (0.50)	27.47 (0.50)	27.53 (0.50)
Missing	12	1260	< 5
White ethnicity % (n/N)			
G0	99% (203/205)	97% (15,175/15,565)	100% (114/114)
G1	96% (346/360)	95% (11,447/12,055)	98% (175/178)
Missing	58	5799	18

**Fig. 1:**
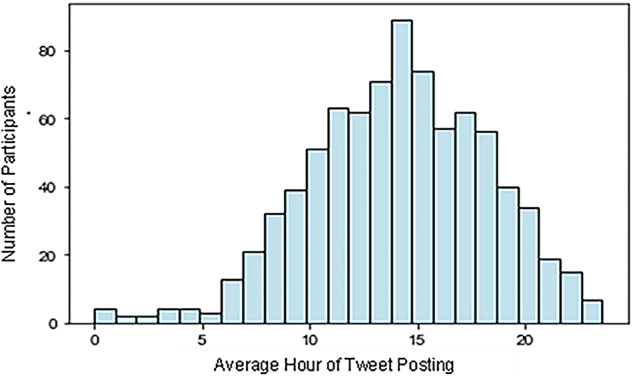
Histogram showing the distribution of average hours Tweets were posted by participants in the 2-weeks before they completed one of the questionnaires.

310 participants were included in the analysis because they posted one or more Tweet in the two weeks before they completed at least one questionnaire. As participants could contribute data from up to five questionnaires, we had 824 responses to analyse. These 310 participants posted a total of 18,288 Tweets. The median number of Tweets posted by a participant in the 2-weeks before they completed a questionnaire was 5 (IQR = 13), with a minimum of 1 and maximum of 1,22. Participants’ SMFQ and GAD-7 scores were positively skewed distribution, whereas WEMWBS scores were normally distributed (Supplementary Materials). The distribution of the average hours participants posted Tweets is shown in Fig. 1. Most participants had average hours of Tweet posting during the daytime.

### Model results

Table 3 shows the results from the adjusted and unadjusted mixed effect models predicting SMFQ, GAD-7 or WEMWBS score from the average hour of Tweet posting. The partial R^2^ scores quantify the amount of variation explained by the average hour of Tweet posting fixed effects, after accounting for the sex and generation of participants. As the unadjusted model does not contain sex and generation terms, the partial R^2^ score is identical to the marginal R^2^ score. The number of observations and unique participants used to fit the models are shown in Table 3, alongside the number of Tweets posted by participants in the two-week periods. All P-values equal to or below 0.001 are shown in bold.

There was good to very strong evidence that the average hour of Tweet posting was associated with SMFQ, GAD-7 and WEMWBS scores. The average hour of Tweet posting explained 0.3% of the variation in SMFQ score, 0.6% in GAD-7 score and 1.8% in WEMWBS score. This association remained after sex and generation terms were added to the models. After taking sex and generation into account, the average hour of Tweet posting explained 0.2% of the variation in SMFQ score, 0.7% in GAD-7 score and 1.9% in WEMWBS score.

**Table 3:** Results from the unadjusted and adjusted models predicting SMFQ, GAD-7 or WEMWBS score from average hour of tweet posting.

Model	SMFQ (depressive symptoms)		GAD-7 (anxiety symptoms)		WEMWBS (wellbeing)	
	Unadjusted	Adjusted	Unadjusted	Adjusted	Unadjusted	Adjusted
Observations	786		801		628	
Participants	302		304		277	
Tweets	17,105		17,598		14,445	
LRT *P*-value	0.002	0.001	< 0.001	< 0.001	< 0.001	< 0.001
Partial R^2^	0.003	0.002	0.006	0.007	0.018	0.019
Marginal R^2^	0.003	0.145	0.006	0.132	0.018	0.065
AIC	7343.7	7297.9	7472.4	7432.3	4226.1	4206.5
BIC	7376.4	7339.9	7505.2	7474.5	4261.6	4251.0

Table 4 shows the relevant results from the models which included an interaction between the average hour of Tweet posting and the demographic covariates. These models were fitted using the same amount of observations, participants and Tweets as in Table 3. The likelihood ratio tests compared the fit of these interaction models to the adjusted model. In these models, the partial *R*^2^ scores are calculated by pooling the amount of variation explained by the circular terms alone and by their interaction with sex and/or generation. In this table, the abbreviation “Gen” refers to generation of participants.

**Table 4:** Results from the interaction models predicting SMFQ, GAD-7 or WEMWBS score from average hour of tweet posting.

Model	SMFQ (depressive symptoms)			GAD-7 (anxiety symptoms)			WEMWBS (wellbeing)		
	Sex & Gen	Sex	Gen	Sex and Gen	Sex	Gen	Sex & Gen	Sex	Gen
LRT *P*-value	< 0.001	0.001	< 0.001	0.008	0.327	0.001	0.620	0.920	0.282
Partial R^2^	0.017	0.008	0.013	0.011	0.008	0.011	0.023	0.020	0.023
Marginal R^2^	0.156	0.148	0.153	0.136	0.132	0.136	0.068	0.065	0.069
AIC	7252.0	7288.1	7261.9	7427.5	7435.7	7422.4	4202.3	4206.6	4202.5
BIC	7331.4	7348.8	7322.6	7507.1	7496.6	7483.3	4282.2	4268.8	4264.7

There was evidence that including terms which assumed an interaction between the average hour of Tweet posting and both covariates improved the fit of models predicting depressive and anxiety symptoms. There was also evidence that only including the generation of participants as an interaction term also improved these models. There was evidence when predicting depressive symptoms that including just sex as an interaction term improved model fit. There was no evidence that including these interaction terms improved the fit of models predicting mental wellbeing. Across all the outcomes, there was much stronger evidence from the LRTs that generation should be included in models as an interaction term, when compared to sex. This is further supported by the partial R^2^ scores, which found that interactions between average hour of Tweet posting and generation explained more of the variation in the outcomes.

### Predicted outcome values

**Fig. 2:**
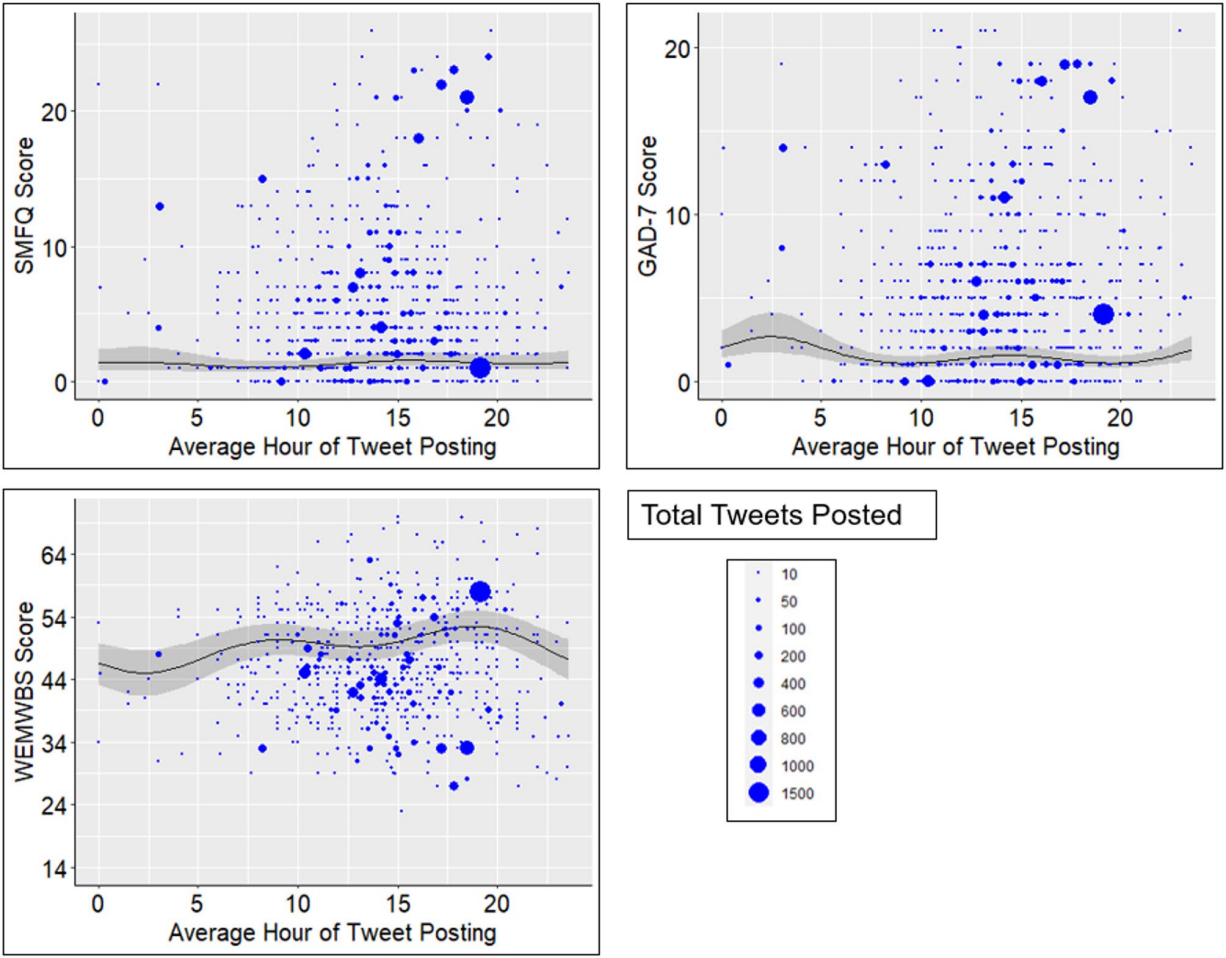
Scatterplots where each dot represents one participant’s SMFQ, GAD-7 or WEMWBS score from a single questionnaire (y-axis), and the average hour they posted Tweets during the 2-weeks before they completed that questionnaire (x-axis). The area of each dot is scaled based on how many Tweets the participant posted during that 2-week period. The line shows the predicted relationship between average hour and the outcome values from the best-fitting mixed effect models, with 95% CI (shaded grey areas).

Based on the results described above, we identified the best performing models for the three outcomes. As there was no evidence that the interactions terms improved the fit of models predicting WEMBWS score, we chose the adjusted model as the best performing, as it outperformed the unadjusted model on all criteria (e.g. lower AIC, lower BIC, higher R^2^). When predicting GAD-7 score, the model with only the generation interaction was the best performing across all criteria. Finally, when predicting SMFQ score the best performing model across all criteria, apart from BIC, was the model with both the sex and generation interaction terms. Figure 2 shows for each average hour of Tweet posting, the outcome values that these best performing models predicted. 95% confidence intervals (CI) for these predicted outcome values are shown, alongside the actual outcome values.

There was evidence that lower WEMWBS and higher GAD-7 scores were predicted for participants with average hours of Tweet posting through the night (23:00 to 05:00). The highest WEMWBS score of 52.47 (95% CI 50.02, 54.93) was predicted for participants with average hours at 18:50 to 18:55, with the lowest score of 44.99 (95% CI 41.36, 48.62) being predicted at 02:20. Using the upper and lower bounds of these predictions’ 95% CI, the smallest possible difference in WEMWBS score was 1.4 and the largest was 13.57. In general, predicted WEMWBS scores from average hours of Tweet posting through the night were around 4 to 8 points lower compared to those during the daytime.

The highest GAD-7 score of 2.68 (95% CI 1.73, 4.13) was predicted when average hour was 2:30, compared to a lowest score of 1.09 (95% CI 0.79, 1.48) at 19.23 to 19.45. From the 95% CI of these predictions, the smallest possible difference in GAD-7 score was 0.25 and the largest was 3.34. In general, predicted GAD-7 scores from average hours through the night were around 1 to 2 points higher compared to those during the daytime.

There was no strong evidence to support a difference in predicted SMFQ score for participants with different average hours of Tweet posting.

### Stratified models

Guided by the results from the interaction models, we produced models stratified by generation to predict SMFQ and GAD-7 score, and a model stratified by sex to predict SMFQ score. The aim of this analysis was to further examine how the sex and age of participants influenced the previously explored relationships. The generation stratified models included sex as a covariate, and the sex stratified contained generation as a covariate. LRTs were used to compare these stratified models to their null models without the average hour terms (Table 5).

**Table 5:** Relevant results from the sex and generation stratified models predicting SMFQ and GAD-7 scores.

Model	SMFQ (depressive symptoms)				GAD-7 (anxiety symptoms)	
	G0	G1	Male	Female	G0	G1
Observations	307	479	320	481	244	284
Participants	113	189	115	189	103	174
Tweets	8909	8196	9236	8362	7663	6782
LRT *P*-value	< 0.001	< 0.001	0.003	< 0.001	< 0.001	< 0.001
Partial R^2^	0.012	0.012	0.009	0.008	0.013	0.006

Across all the stratified models there was good to very strong evidence that the average hour of Tweet posting was associated with SMFQ and GAD-7 score. After stratifying by generation and sex, the amount of variation explained by the average hour of Tweet posting (partial R^2^) increased. There was little to no difference in partial R^2^ score across the strata when predicting SMFQ score. In contrast, the average hour explained more than double the variation in GAD-7 score in the older G0 participants. This amount of variation explained (1.3%) was also larger than the partial R^2^ score observed in the unstratified model (0.6%). Comparisons across strata of the predicted outcome values from these models can be found in Supplementary Materials. While there are no large differences in predicted outcome values between the models being compared, the female and G0 models appear to show more variation across the range of average hours.

## Discussion

We investigated the relationship between the timing of Twitter activity and mental health and wellbeing. We found evidence that the average hour participants posted Tweets over a 2-week period was associated with self-reported concurrent depressive symptoms, anxiety symptoms and mental wellbeing. This association remained even after the inclusion of sex and age covariates as model terms.

There was evidence that the average hour at which participants posted Tweets was associated with their mental wellbeing. Average hour explained 1.9% of the variation in mental wellbeing, a larger amount than for depressive and anxiety symptoms. Participants who on average posted Tweets through the night (i.e. 23:00 to 05:00) had worse mental wellbeing (approximately four to eight points lower) compared to those who, on average, Tweeted during the day. Previous research has found that for an individual difference in WEMWBS score to be important, it must be above the threshold of 3 points^54,55^. Our observed difference was above this threshold, providing evidence for a meaningful association between the average hour of Tweet posting and mental wellbeing. No previous study has specifically explored this relationship between timing of social media or technology use and mental wellbeing. Actively using Twitter during the night could disrupt and delay sleep, reducing its quality and quantity^20,56^. This could harm mental wellbeing, and explain the results we found. Future studies should specifically explore this mechanism by investigating whether measures of sleep quality or quantity mediate the relationships we observed.

It is useful to contextualise the amount of variation in mental wellbeing explained by the average hour of Tweet posting by comparing it to results found using other potential predictors in previous studies. An Australian cohort study (*N* = 1399) investigated which relevant factors were associated with wellbeing measured using a self-reported wellbeing scale^57^. Compared to average hour of Tweet posting in our study, risk factors that explained a similar amount or less of the variation in wellbeing were fruit and vegetable intake (1.97%), sleep hours per night (1.3%) and smoking status (1.1%)^57^. A larger study (*N* = 12,826) of Canadians measured life satisfaction^58^. The amount of variation in life satisfaction explained by average hour of Tweet posting exceeded frequency of physical exercise (1.8%), disability status (1.3%), and neighbourhood safety (0.9%)^58^. A study using data from a cohort of American children and adolescents (*N* = 74,814) measured wellbeing using questions capturing mental health and suicide ideation^10^. Compared to average hour in our study, risk factors that explained a similar amount of the variation in wellbeing were binge drinking (2.1%) and smoking marijuana (1.8%)^10^. These comparisons are not perfect, as our sample is based in a different population and used a different measurement of wellbeing. However, they provide necessary context regarding the meaningfulness of our association between average hour and mental wellbeing.

Our findings support the push for interventions aiming to reduce the amount of time individuals use social media at nighttime. These interventions could be targeted towards entire populations (e.g. curfews for adolescents, TikTok’s “Wind Down” tool), allow individuals to control their own nighttime use, or provide social media users information about the potential risks of nighttime use. Whilst observational studies like our can indicate the potential need for such interventions, their introduction should ideally be supported by studies specifically exploring their impact. One such example is the SmartSleep Experiment, in which the impact of a national mass media campaign focusing on smartphone behaviours and sleep was evaluated^59^. This campaign involved content on radio programmes, websites and social media pages^59^. 8,894 Danish adults provided measures of nighttime smartphone use before and after this campaign^59^. Prior to the campaign, 4926 participants reported using their smartphone during sleep hours, and at follow-up 598 of these participants reported that they were using their smartphones less at this times^59^. Interventions aiming to reduce nighttime use of social media could also be explored through randomized controlled trials. For example, a study evaluated the impact of smartphone users (*N* = 70) following ten steps aimed at reducing problematic use of social media (e.g. keeping phone of silent and out of reach at bedtime, disabling non-essential notifications)^60^. This intervention was compared to a control group, who only monitored their screen time^60^. Whilst there was evidence that this intervention reduced daily screen time and self-reported problematic use of social media, there was no evidence for a change in sleep quality^60^. Other social media-based interventions, for example the TikTok “Wind Down” tool could also be evaluated using such randomized controlled trials.

As with mental wellbeing, there was robust evidence that the average hour a participant posted their Tweets was associated with their depressive and anxiety symptoms. However, less of the variation in depression (0.2%) and anxiety (0.7%) was explained. Even the best performing model found little to no variation in predicted depressive symptoms depending on the average hour a participant posted their Tweets. Participants with average hours of Tweet posting through the night were predicted to have slightly more anxiety symptoms than those with average hours during the rest of the day. However, this difference (around 1 to 2 points) was below the threshold that research previous has found to be considered a minimum clinically important difference (4-points)^61^. As a result, our study found no evidence to support a meaningful clinical association between the time of day that individuals use Twitter and their depressive and anxiety symptoms.

Three previous studies have found evidence that the relationship between nighttime social media or technology use and anxiety is either absent or weak^21,22,38^. For example, one study measured self-reported social and generalized anxiety, and found no evidence for a relationship with the proportion of Tweets posted at nighttime^38^. Our findings of a weak and not clinically meaningful relationship between average hour of Tweet posting and anxiety generally supports these previous studies. However, our findings when predicting depressive symptoms contrasted with previous studies. Compared to anxiety, more studies of nighttime social media or technology use have examined depressive symptoms as an outcome. Previous studies have generally found evidence for a weak positive correlation between self-reported nighttime social media or technology use and depressive symptoms^21,22,24,62^. Earlier research which also used data directly collected from Twitter generally found evidence that depressed participants were more likely to post Tweets at nighttime^33,34,36,38^. Methodological differences between our study and previous studies, which used either survey-reported social media use or actual Twitter data, could explain the differences in results. Self-reported measures of social media use are subject to measurement error. If participants with higher depressive symptoms are more likely to overestimate their nighttime social media use, this would lead to an exaggerated association. Compared to previous studies using Twitter data, we used a new approach to characterise the time of day our participants posted Tweets. Unlike previous Twitter studies, we also produced models and analyses which were weighted by the total number of Tweets each participant posted. Furthermore, our sample was older than previous studies. Unlike most studies using Twitter data, we treated depression and anxiety as continuous, rather than binary, variables. Finally, we restricted our analyses to Tweets posted 2-weeks before a self-reported measure of depression was provided. Previous Twitter studies use Tweets posted over a much larger period of time. Tweets posted close to the completion of these questionnaire measures may be a more accurate reflection of a participant’s mental health at that time.

The best performing models predicting depressive and anxiety symptoms included an interaction between the average hour of Tweet posting and the demographic characteristics of participants. Including these interactions increased the amount of variation explained in depressive and anxiety symptoms. There was evidence for an interaction between average hour and the age of participants in models predicting depressive and anxiety symptoms. However, there was only evidence for an interaction between average hour and sex in the model predicting depressive symptoms. Across all mental health variables, there was much strong evidence for an interaction between average hour and age, compared to average hour and sex.

Where indicated by evidence of an interaction effect, stratifying models based on the age and sex of participants revealed interesting findings. Stratification generally increased the amount of variation in depressive and anxiety symptoms explained by average hour of Tweet posting. There was stronger evidence for a relationship between average hour of Tweet posting and depressive symptoms in females, and average hour and anxiety symptoms in older participants. These results suggest that the demographic characteristics of participants may influence the relationship between the timing of Twitter activity and depressive and anxiety symptoms. We are the first study to explore these interactions, and further studies are needed to examine how the characteristics of participants influence the relationship between social media behaviour and mental health. This could help generate more nuanced results regarding the individuals who are most at risk of any harms of social media, or who may be most benefited. Furthermore, such research could inform whether interventions should be specifically targeted towards those particularly at risk of harm from nighttime social media use. For example, if the relationship between nighttime Twitter activity and mental health is stronger in female users, as our study suggests, educational campaigns on the risk of nighttime use could be advertised on social media content that these users are more likely to see.

Unlike most previous studies, we measured social media behaviour using data directly from Twitter and not through self-reported surveys. We used validated and reliable self-report questionnaires to estimate the mental health status of participants. We are also the first study in this field of research to include mental wellbeing as an outcome. In contrast to measures of depression and anxiety, mental wellbeing captures some of the more positive aspects of mental health, which are often overlooked in previous social media research^35^. We also controlled for sex and age in our models, which increases confidence that our observed relationships were not confounded by the demographic characteristics of our participants. To quantify the time of day that our participants posted their Tweets over a two-week period, we used a novel approach for this field. We modelled the hour each Tweet was posted as a circular variable ranging from 0 to 2π. We then calculated the circular mean of this new variable. This approach has the advantage of not grouping Tweets into large daytime and nighttime windows, and thus can differentiate between behaviours at a finer scale. For example, a participant who posted all their Tweets between 21:00 to 23:00 will have a different average hour than a participant with Tweets all posted between 02:00 and 05:00. As a result, our approach can quantify the time of day a participant posted their Tweets into a single measure, without losing key information that can differentiate behaviour between participants. Our study also has some limitations that should be considered when interpreting the findings. First, timing of Twitter activity was only measured using the hour at which participants posted Tweets, which is an active and public behaviour. To mitigate the risk of identification of ALSPAC participants, our study measured the time a Tweet was posted in one-hour time windows (e.g. a Tweet posted at 21:08 is labelled as 21:00). This small reduction in resolution is unlikely to have affected the results of this study. We also did not incorporate sleep variables into our models, because there were not appropriate measures of sleep quality or quantity collected within the questionnaires we used. In addition, our participants were nearly all white, more likely to be female and entirely comprised of adults. This is not representative of the demographics of all ALSPAC participants who reported having a Twitter account (*n* = 2,294) and previous UK surveys of Twitter users^63–65^. We also used Twitter and survey data which was collected during the COVID-19 pandemic. Patterns of mental health and social media use may have been different during the pandemic, compared to times outside of it. For example, previous research has found evidence that ALSPAC participants reported higher GAD-7 scores and lower WEMWBS score during the pandemic^66^. No study has explored whether the time-of-day people used social media changed during the pandemic, although one found that their 1310 participants reported using digital media more frequently before bedtime during lockdown^67^. Whilst our study should be replicated using non-pandemic data, researching these relationships during a period in which both mental health problems and nighttime use of social media may have been exacerbated is important.

Our study applies a new approach to understand the relationship between timing of social media activity and mental health. We found evidence to support that use of Twitter through the night has a meaningful association with worse mental wellbeing, but not with symptoms of depression or anxiety. The relationship between the timing of Twitter activity and mental wellbeing was comparable to factors such as binge drinking, frequency of exercise and sleep hours per night, as found in previous studies. This research is likely to be relevant evidence for legislation aiming to improve the safety of social media, as it highlights the potential impact of a very specific problematic behaviour. These behaviours could be targeted in interventions, with the objective of reducing the amount they are performed. Our findings suggest that social media use through the night is a behaviour such interventions could target.

## Supplementary Information

Below is the link to the electronic supplementary material.

Supplementary Material 1

## Data Availability

The dataset analysed in this study is not publicly available, as the informed consent obtained from ALSPAC participants does not allow data to be made freely available through any third party maintained public repository. ALSPAC data can be made available on request to the ALSPAC, with full instructions for applying for data access found here: http://www.bristol.ac.uk/alspac/researchers/access/.

## Abbreviations

ALSPAC: Avon longitudinal study of children and parents
SMFQ: Short Mood and Feelings Questionnaire
GAD-7: Generalised Anxiety Disorder 7
WEMWBS: Warwick Edinburgh Mental Wellbeing Scale
LRT: Likelihood ratio test
EQ: Equation
CI: Confidence intervals
