# Supplementary material for: Active night-time tweeting is associated with meaningfully lower mental wellbeing in a UK birth cohort study
Source: Sci Rep. 2025 Oct 9;15:34301. doi: 10.1038/s41598-025-14745-y (PMC12511309; doi:10.1038/s41598-025-14745-y)
Supplement: Supplementary file 1 — Supplementary Material 1 [file 41598_2025_14745_MOESM1_ESM.docx]

## Supplementary Materials

### Circular Mean Calculation

The hour each Tweet was posted at was treated as a circular variable, which can be represented on the circumference of the unit circle (Supplementary Figure 1) [1]. Each hour is assigned a point *z* on the circumference of this circle, starting anticlockwise from 00:00 at the origin (Supplementary Figure 1). Each circular observation *z* can be represented as a vector *x,* connecting the centre of the circle to the point on the circumference [1]. This vector generates an angle $\theta$*,* measuring anticlockwise from 00:00 [1]. These angles range from 0 to 2*π*. For example, a Tweet posted at 06:00 will correspond to the angle *π/*2. The vector *x* is comprised of two components, *cos*$\theta$*,* and *sin*$\theta$*,*. These components are the basis for the calculation of a circular mean angle.


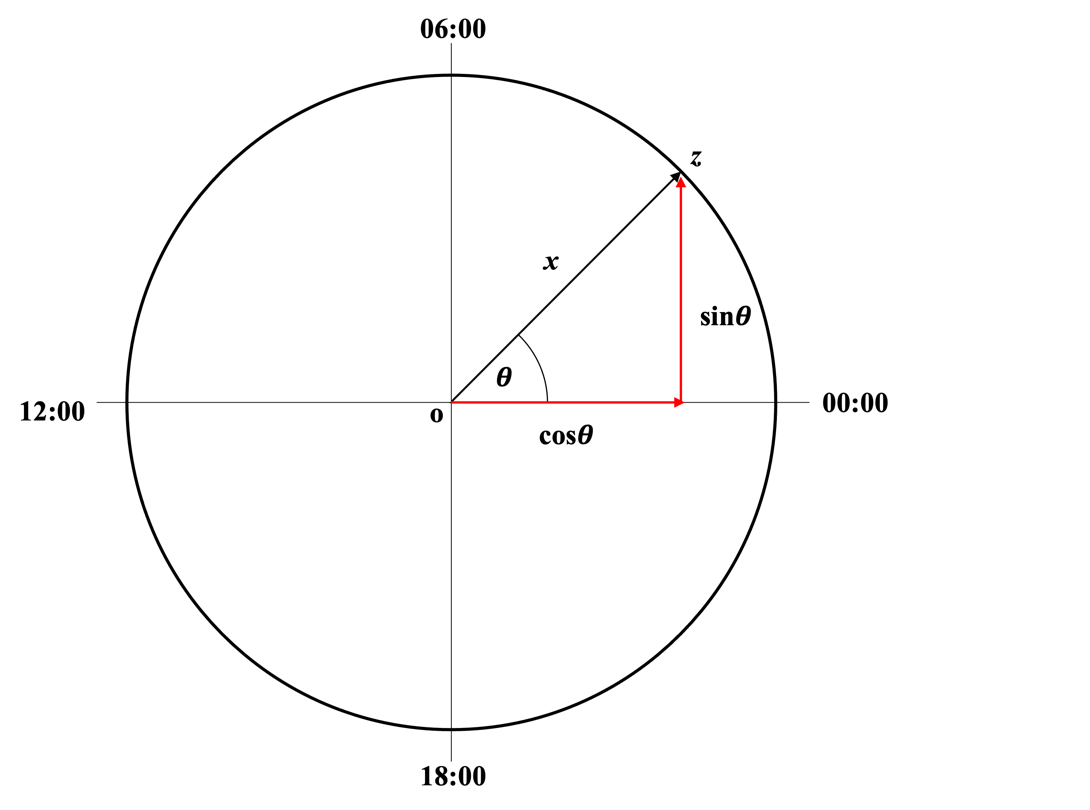


### Supplementary Figure 1: Circular variable representation of the hour a Tweet is posted at

For each participant, the mean angle $\bar{\theta}$ for their Tweets was calculated using the following equations [1]:


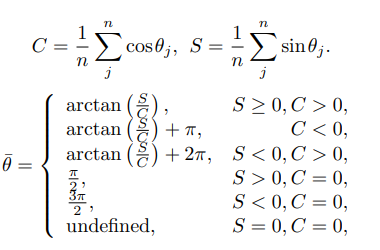


$\bar{\theta}$ is a continuous variable, ranging from 0 to 2*π*. This can be converted into a mean hour of Tweet posting, using the equation:


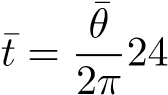


### Distributions of SMFQ, GAD-7 and WEMWBS scores

Supplementary figure 2 shows the distributions of SMFQ, GAD-7 and WEMWBS scores in the dataset. Participants can contribute up to five observations of SMFQ or GAD-7 scores, and up to four observations of WEMWBS scores.


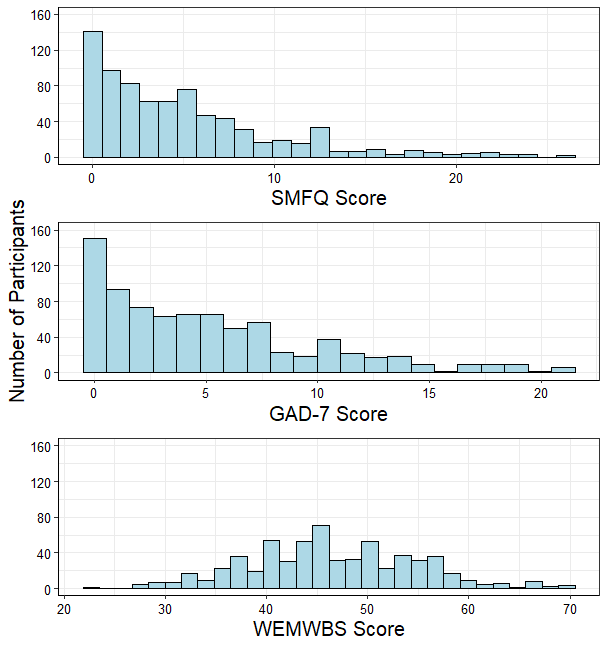


Number of Observations

Supplementary Figure 2: Histogram of SMFQ, GAD-7 and WEMWBS score for observations across all five questionnaires in the dataset.

Supplementary Table 1 shows the descriptive statistics from participants’ responses to these three outcomes.

Supplementary Table 1: Descriptive statistics for participants’ responses to the SMFQ, GAD-7 and WEMWBS surveys across the five COVID-19 questionnaires.

| Measure | Mean (SD) | Median (IQR) | Range |
| --- | --- | --- | --- |
| SMFQ | 5.00 (5.18) | 4.00 (6.00) | 0.00 – 26.00 |
| GAD-7 | 5.04 (4.96) | 4.00 (6.00) | 0.00 – 21.00 |
| WEMWBS | 46.53 (8.34) | 46.53 (11.25) | 23.00 – 70.00 |

### Predicted Outcome Values from the Sex and Generation Stratified Models

Supplementary Figure 3 shows the predicted outcome values from the male and female models predicting depressive symptoms.
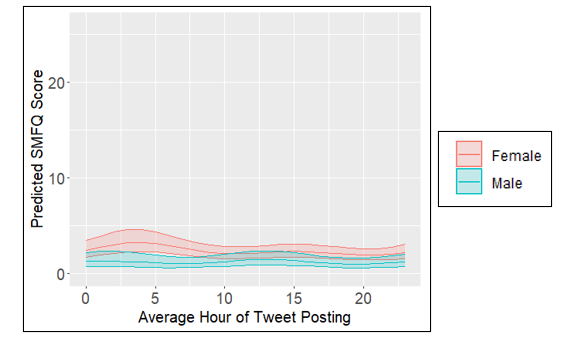


Supplementary Figure 3: Predicted SMFQ scores across the range of average hours of Tweet posting for the female and male stratified mixed effect models. Shaded areas show 95% confidence intervals.


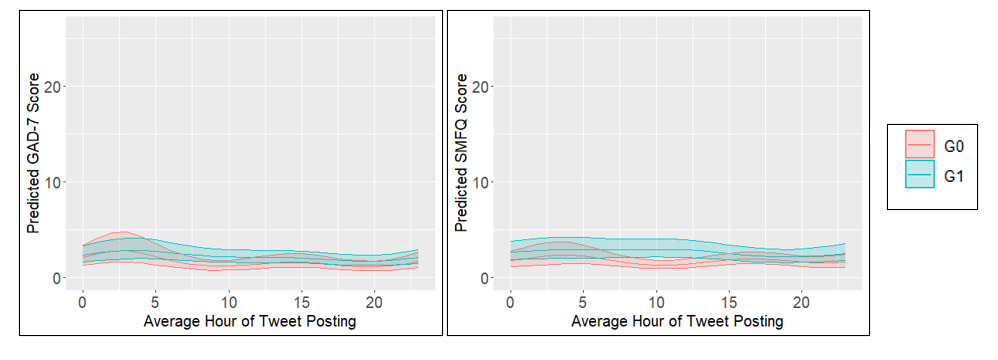
Supplementary Figure 4 shows the predicted outcome values from the G0 and G1 models predicting depressive and anxiety symptoms.

Supplementary Figure 4: Predicted SMFQ and GAD-7 scores across the range of average hours of Tweet posting for the G0 and G1 stratified mixed effect models. Shaded areas show 95% confidence intervals.

## References:

1. Pewsey A, Neuhauser M, and Ruxton GD. Circular statistics in r. 2013.
